# Acupuncture for polycystic ovary syndrome: An overview of a protocol for systematic reviews and meta analyses

**DOI:** 10.1097/MD.0000000000024218

**Published:** 2021-01-22

**Authors:** Zaibo Liao, Huaying Fan, Huayu Fan, Xiaohua Chen

**Affiliations:** a The Center of Gerontology and Geriatrics, West China Hospital, Sichuan University / West China School of Nursing, Sichuan University; b College of Acupuncture and Tuina, Chengdu University of Traditional Chinese Medicine; c Respiratory Failure Center and Lung Transplant Unit, Sicuhan Province Hospital; d Department of Central Transportation Center, West China Hospital, Sichuan University, Chengdu city, Sichuan Province, China.

**Keywords:** acupuncture, overview, polycystic ovary syndrome, protocol

## Abstract

**Background:**

As the results of previous systematic reviews and meta-analyses on acupuncture for polycystic ovary syndrome (PCOS) have provided inconsistent evidence. This overview of systematic reviews (SRs) and meta-analyses will aim to critically appraise the methodology and reporting quality of the relevant SRs and meta-analyses with the aim of identifying whether acupuncture could provide an effective treatment for patients with PCOS or not.

**Methods:**

Electronic databases including MEDLINE via Ovid, EMBASE, PubMed, Cochrane library, China National Knowledge Infrastructure (CNKI), Chinese Scientific Journal Database (VIP database), and Wanfang Database will be searched for related SRs and meta-analyses from inceptions to the search date without language restrictions. Two reviewers will independently select SRs and meta-analyses and collect related data, and a third reviewer will be introduced if any disagreement happened during the assessing. The Preferred Reporting Items for Systematic Review and Meta-Analyses (PRISMA) and the latest Assessment of Multiple Systematic Reviews 2 (AMSTAR2) checklists will be employed to evaluate the reporting and methodology quality.

**Results:**

This overview will be published in a peer-reviewed journal.

**Conclusion:**

This overview will identify related SRs and meta-analyses of acupuncture for treating PCOS.

**Ethics and dissemination:**

Ethics approval and patient consent are not required as this study is an overview based on published systematic reviews and meta-analyses.

## 1 Introduction

Polycystic ovary syndrome (PCOS) is one of the most common cause of an ovulatory infertility conditions in clinic, which is characterized by ovulatory dysfunction, hyperandrogenism, and polycystic ovaries.^[1]^ It is estimated that PCOS prevalence has varied from 5% to 15% among women of reproductive age.^[2]^ PCOS can result in profound, long-term physical, and mental health consequences.^[3,4]^ In addition to an ovulatory infertility, women suffering from PCOS have an increased risk of miscarriage, pregnancy complications, and metabolic abnormalities such as obesity, hypertension, and non-alcoholic fatty liver disease.^[5–8]^ The cause of PCOS is genetic related,^[9]^ and environmental factors such as prenatal exposure to androgens may also play a role in the etiology of PCOS.^[10]^

Currently, pharmacological therapies for PCOS include clomiphene, which is considered as the first-line treatment to induce ovulation in women with PCOS.^[11]^ However, clomiphene turned out to have a high failure rate of without ovulation, a relatively low cumulative live birth rate, and a high multiple-pregnancy rate.^[11]^ Other pharmacological treatments such as oral contraceptives are used, but it is reported that long-term use of oral contraceptives in women with PCOS may lead to the development of obesity and metabolic abnormalities.^[12]^ Given the fact that clomiphene and oral contraceptives have some side effects during the treatment of PCOS, alternative therapies have become an important supplement for PCOS treatment, among which acupuncture has played an important role. Acupuncture, as an integral part of traditional Chinese medicine (TCM), has gained increased popularity in clinical treatment of PCOS.^[13]^ Although the efficacy of acupuncture for treating PCOS has been proved in clinical practice, some researchers suggest that acupuncture works through placebo effect instead of its real therapeutic effect.^[14,15]^ Several systematic reviews (SRs) were carried out to investigate the effect of acupuncture on PCOS, yet gaining inconsistent conclusions.^[16–19]^ Therefore, it is necessary for us to undertake an overview of SRs and meta-analyses to evaluate the methodology and reporting quality of the SRs and meta-analyses.

## 2 Methods

### 2.1 Protocol and registration

This protocol will be undertaken in accordance with the Preferred Reporting Items for Systematic Reviews and Meta-Analysis Protocols (PRISMA-P),^[20]^ and the study has been registered on OSF platform (https://osf.io/registries) with a registration NO. 10.17605/OSF.IO/Z3BPV.

### 2.2 Ethics

Ethics application was not required as this study is based on published SRs and meta-analyses.

### 2.3 Information sources and search strategy

The following 7 electronic databases will be searched for relevant SRs and meta-analyses from their inception to 2019, irrespective of language and publication status: MEDLINE via Ovid, EMBASE, PubMed, Cochrane library, China National Knowledge Infrastructure (CNKI), Chinese Scientific Journal Database (VIP database), and Wanfang Database. The detailed search strategy in PubMed is given in Table 1.

**Table 1:** Search strategy draft.

Number	Entry terms
#1	Ovary syndrome, polycystic [Title/Abstract]
#2	Syndrome, polycystic ovary [Title/Abstract]
#3	Stein-Leventhal syndrome [Title/Abstract]
#4	Stein Leventhal syndrome [Title/Abstract]
#5	Syndrome, Stein-Leventhal [Title/Abstract]
#6	Sclerocystic ovarian degeneration [Title/Abstract]
#7	Ovarian degeneration, sclerocystic [Title/Abstract]
#8	Sclerocystic ovary syndrome [Title/Abstract]
#9	Polycystic ovarian syndrome [Title/Abstract]
#10	Ovarian syndrome, polycystic [Title/Abstract]
#11	Polycystic ovary syndrome [Title/Abstract]
#12	Sclerocystic ovaries [Title/Abstract]
#13	Ovary, sclerocystic [Title/Abstract]
#14	Sclerocystic ovary [Title/Abstract]
#15	or/#1–#14
#16	acupuncture [MeSH Terms]
#17	acupuncture analgesia [MeSH Terms]
#18	acupuncture analgesia [MeSH Terms]
#19	acupuncture therapy [MeSH Terms]
#20	acupuncture, ear [MeSH Terms]
#21	or/#16–#20
#22	systematic review [Title/Abstract]
#23	meta-analysis [MeSH Terms]
#24	meta-analysis [Title/Abstract]
#25	or/22–24
#26	#15 and #21 and #25

### 2.4 Eligibility criteria

The PICOS (participant, intervention, comparison, and study design) principle will be utilized in this study.

#### 2.4.1 Study design

All published SRs and meta-analyses of randomized controlled trials (RCTs) or quasi-RCTs involving acupuncture for PCOS will be included without restriction on language or publication type. Non-RCT SRs and meta-analyses, review comments, overview of SRs, editorials, and guidelines will be excluded.

#### 2.4.2 Participants

Female patients of reproductive age diagnosed with PCOS will be included.

#### 2.4.3 Interventions and comparison

The intervention will include acupuncture as manual acupuncture, electroacupuncture, and warm acupuncture. SRs and meta-analyses with acupuncture combined therapy or acupuncture related treatment like point injection, laser acupuncture, transcutaneous electrical nerve stimulation (TENS), cupping, or blood-letting, will be excluded.

The comparison treatments with sham acupuncture, western medicine, placebo, no treating/waiting list will be included. SRs and meta-analyses with the control group compared different forms of acupuncture treatment will be excluded.

#### 2.4.4 Outcomes

The primary outcomes of this overview will be ovulation induction. Secondary outcomes will be luteinizing hormone/follicle-stimulating hormone, menstrual frequency, birth live, pregnancy, conception, biochemical assessments, anthropometry. Adverse events such as acupuncture fainting, needle twisting and breaking, bleeding, and organ injury will also be taken into account as safety measurement.

### 2.5 Selection of studies and data extraction process

Two reviewers (HF and HF) will respectively screen the study titles and abstracts to identify potentially eligible SRs and meta-analyses according to the inclusion criteria. The full texts reviews will be obtained and independently screened before final inclusion. If a disagreement on the inclusion of a review cannot be resolved, a third reviewer (XC) will make the final decision after discussion.

**Figure 1:**
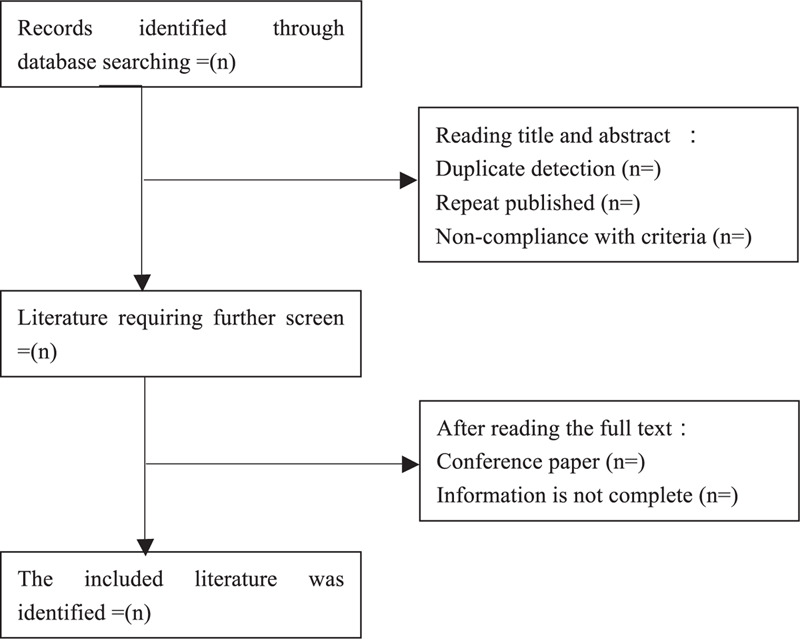
Flow chart of literature selection.

Two reviewers (ZL and HF) will independently extract the data of all eligible SRs and meta-analyses by employing the specially designed extraction forms. The following items will be included in the data extraction form: first author, year of publication, country, number of RCTs enrolled, quality assessment tool for RCTs, and characteristics of interventions in control and treatment groups, outcome measures, data synthesis methods, main results, and conclusions. The final extracted data will be cross-checked by the two extractors, and any disagreement regarding the extracted data will be discussed with and adjudicated by a third reviewer (XC). If any data are insufficient or unclear, the first or corresponding author for the study concerned will be contacted via e-mail or telephone to provide additional information. The flow chart is displayed in Fig. 1.

### 2.6 Quality assessment

The assessment of Multiple Systematic Reviews 2 (AMSTAR2) will be utilized to assess methodological qualities of the included SRs and meta-analyses. AMSTAR is a quality assessment tool for critically assessing the quality of RCTs, while AMSTAR2, which is an update of AMSTAR, can be used to appraise SRs and meta-analyses of intervention trials including both RCTs and non-RCTs.^[21]^ There are 16 items in AMSTAR2. Items 2, 4, 7, 9, 11, 13, and 15 are consider to be more critical than other items, which may affect the production of the systematic review and the validity of the results. The main rule for rating overall quality in the results of SRs and meta-analyses as follows: SRs and meta-analyses with no or 1 noncritical weakness will be rated as high; with >1 noncritical weakness will be rated as moderated; with 1 critical flaw with or without noncritical weakness will be rated as low; with >1 critical flaw with or without noncritical weakness will be rated as critically low.

Two reviewers (ZL and HF) will independently assess the methodological quality of each included SR and meta-analysis by employing the AMSTAR2. Any disagreement during the assessment will be discussed or solved by a third reviewer (XC).

Preferred Reporting Items for Systematic reviews and Meta-Analyses (PRISMA) will be applied to evaluate the report quality of included SRs and meta-analyses. There are 27 items used to assess whether the reports are standardized or not. Discussion and a third reviewer will be introduced when confronted with disagreement during the assessing process (XC).

### 2.7 Data synthesis

General characteristics of the included systematic reviews will be summarized and described. PRISMA and AMSTAR2 will be utilized to assess each review to show the risk of bias, and the total percentage and the 95% confidence interval of each item will be calculated.

## 3 Discussion

With an increasing number of SRs and meta-analyses on acupuncture for treating PCOS in recent years, we would like to figure out whether those SRs and meta-analyses of acupuncture for PCOS have high methodological quality and reporting quality. It is known that systematic reviews and meta-analyses with high quality can help policy-makers or clinical physicians to make better choice and clinical practice, therefore, an overview of SRs and meta-analyses is essential to be done by using AMSTAR2 and PRISMA tools to provide stronger evidence for acupuncture for treating PCOS.

## Author contributions

**Conceptualization:** Zaibo Liao, Huaying Fan.

**Data curation:** Zaibo Liao, Huayu Fan.

**Formal analysis:** Huaying Fan.

**Investigation:** Huaying Fan, Huayu Fan, Xiaohua Chen.

**Methodology:** Huayu Fan, Xiaohua Chen.

**Project administration:** Huaying Fan, Xiaohua Chen.

**Supervision:** Xiaohua Chen.

**Validation:** Xiaohua Chen.

**Writing – original draft:** Zaibo Liao, Huaying Fan.

**Writing – review & editing:** Xiaohua Chen.
